# Enhanced Performance of Fly Ash-Based Supports for Low-Cost Ceramic Membranes with the Addition of Bauxite

**DOI:** 10.3390/membranes11090711

**Published:** 2021-09-15

**Authors:** Wan Fan, Dong Zou, Jingrui Xu, Xianfu Chen, Minghui Qiu, Yiqun Fan

**Affiliations:** State Key Laboratory of Materials-Oriented Chemical Engineering, College of Chemical Engineering, Nanjing Tech University, Nanjing 211816, China; fanwan@njtech.edu.cn (W.F.); zoudong0368@gmail.com (D.Z.); jingruixu@njtech.edu.cn (J.X.); chenxianfu@njtech.edu.cn (X.C.); qiumh_1201@njtech.edu.cn (M.Q.)

**Keywords:** low-cost ceramic membranes, fly ash, ceramic supports, bauxite, acid/alkali corrosion resistance

## Abstract

Support is a necessary foundation for ceramic membranes to achieve high performance. Finding the optimum balance between high performance and low cost is still a significant challenge in the fabrication of ceramic supports. In this study, low-cost fly ash-based ceramic supports with enhanced performance were prepared by the addition of bauxite. The pore structure, mechanical strength, and shrinkage of fly ash/bauxite supports could be tuned by optimizing the bauxite content and sintering temperature. When the sintering temperature and bauxite content were controlled at 1300 °C and 40 wt%, respectively, the obtained membrane supports exhibited a high pure water permeance of approximately 5.36 m^3^·m^−2^·h^−1^·bar^−1^ and a high bending strength of approximately 69.6 MPa. At the same time, the optimized ceramic supports presented a typical mullite phase and excellent resistance to acid and alkali. This work provides a potential route for the preparation of ceramic membrane supports with characteristics of low cost and high performance.

## 1. Introduction

Ceramic membranes have been widely used in water treatment owing to their distinctive characteristics, such as high flux, antifouling properties, long life, and flexibility of operation [1,2,3,4,5]. Ceramic membranes generally consist of a support, an intermediate layer, and separation layers [6]. Among these, the support usually occupies more than 95% of the volume of the membrane element; therefore, it is the main contributor to the strength of the ceramic membrane. In addition, a high-performance support is a prerequisite for ensuring membrane permeability. On the one hand, the mass transfer resistance through an asymmetric ceramic membrane with a macroporous support is significantly lower than that of a symmetric membrane. On the other hand, a uniform pore size distribution of ceramic support facilitates the deposition of the subsequent intermediate and separation layers and allows for a reduction in thickness. In summary, the support is a necessary foundation for ceramic membranes to achieve high performance.

Conventional high-performance ceramic membrane supports are usually fabricated from high-purity Al_2_O_3_ powders and sintered at temperatures above 1700 °C [7,8,9]. The high cost of raw materials and energy-consuming fabrication results in expensive ceramic membranes, which in turn restricts their promotion and application especially in cost-sensitive fields. Therefore, the preparation of low-cost ceramic membrane supports has attracted extensive attention in recent years [10,11]. These are usually fabricated from inexpensive raw materials that can be easily sintered, such as kaolin [12,13,14,15], bauxite [16,17], clay [18,19], pozzolan [20], and fly ash [21,22,23,24,25]. Fly ash is an industrial waste generated by coal-fired power plants [26], and it contains mainly Al_2_O_3_, SiO_2_, and other metal oxides. Thus, using fly ash as a raw material to prepare ceramic supports can also reduce the material cost. Zong et al. [24] prepared porous ceramics using high-alumina fly ash microbeads as raw materials via a solid-phase sintering method at 1170 °C. The porosity and bending strength of the obtained ceramics were 49.21% and 12.88 MPa, respectively, and pore sizes ranged from 5 to 50 μm. Jedidi et al. [25] used fly ash as a raw material to prepare tubular ceramic supports. After being sintered at 1125 °C, the average pore diameter of the obtained ceramic supports was approximately 4.5 μm and the porosity was 51%. 

However, fly ash has a wide particle size distribution and low melting temperature, which hinders the control of the sintering process and the obtainment of a macroporous structure with uniform pore size distribution and high porosity. Moreover, the consistency and composition of fly ash are significantly affected by the source and even the season. In recent years, some researchers have tried to adjust the composition of fly ash and control the porous structure of supports through doping methods. Liu et al. [27] prepared porous ceramic supports by adding 28.43 wt% dolomite to fly ash, this resulted in an increase in ceramic porosity from 25.2% to 46.5% and in a bending strength of 35 MPa. Li et al. [28] prepared ceramics reinforced with mullite whisker using fly ash as the raw material, Al_2_O_3_ as the aluminum source, and AlF_3_ as the mullite whisker promoter. After sintering at 1200 °C, the bending strength of the ceramic supports was 59.1 MPa and the porosity was 26.8%. In our previous work [29], fly ash powders were employed to prepare ceramic supports for microfiltration membranes. The addition of mullite fiber to fly ash reduced the shrinkage and improved the permeance and bending strength of the ceramic supports.

As evidenced above, most of the materials added to fly ash are rich in aluminum. The reason for using these components is that the percentage of mullite in the obtained ceramics can be increased by introducing an additional source of aluminum to combine with the free SiO_2_ in fly ash [30]. Mullite is one of the most important oxide materials for conventional ceramics, and it is more stable in the binary systems of SiO_2_ and Al_2_O_3_ [31,32]. Therefore, alumina or aluminum-rich materials are added during the preparation of fly ash supports to react with excess SiO_2_ and produce secondary mullite, thereby enhancing the resistance of the ceramic under harsh conditions [33].

In this study, low-cost ceramic membrane supports were fabricated from industrial waste fly ash and bauxite was employed as an aluminum source. The effects of bauxite content and sintering temperature on the structure and performance of the prepared supports were systematically investigated by taking into account porosity, pore size, bending strength, pure water permeance, and micromorphology. In addition, the resistance of the optimized ceramic membrane supports to acid and alkali was characterized using H_2_SO_4_ and NaOH solutions at 100 °C and the bending strength before and after treatment was measured.

## 2. Experimental

### 2.1. Raw Materials

The fly ash powders used in this study were purchased from a power plant in Hebei Province, China, whereas bauxite powders were purchased from Shijiazhuang, Hebei, China. These materials were used as received, without purification. Glycerol (Sigma–Aldrich, Burlington, MA, USA) and polyvinyl alcohol (PVA; Sigma–Aldrich, Burlington, MA, USA) were used as binders to obtain fly ash supports. The pure water used in this work was deionized water (conductivity < 5 µS·cm^−1^).

### 2.2. Preparation of Fly Ash Support

First, a certain amount of bauxite was added to the fly ash at ratios ranging from 0.2:1 to 1:1. Then, solutions of glycerol and PVA (10 wt% in water) were added in the respective amounts of 4 and 3 wt% to the total mass of bauxite and fly ash. Glycerol acts as a lubricant to make the powder particles mix more evenly. PVA acts as a binder to improve the strength of the green support. Subsequently, the powder and additives were mixed well in a mortar and then shaped using the dry press molding method to form disk supports (30 mm in diameter and 2.8 mm in thickness) and rectangular column supports (50 mm × 6.1 mm × 5.9 mm). The obtained green samples were sintered at different temperatures varying from 1150 to 1300 °C at an interval temperature of 50 °C. During the sintering process, the holding time was 2 h, and the heating rate was 2 °C/min. Samples were generically denoted as Ax-y, where x indicates the mass of bauxite added to fly ash (100 g), and y is the sintering temperature (e.g., a sample without bauxite doping sintered at 1150 °C would be A0-1150; a sample with 40 g bauxite per 100 g fly ash sintered at 1250 °C would be A40-1250). The detailed compositions of the samples are presented in Table 1.

### 2.3. Characterization

The fly ash and bauxite powders were characterized using field-emission scanning electron microscopy (FESEM, S-4800, Hitachi, Japan). The particle sizes of fly ash and bauxite were measured using a laser particle sizer (Mastersizer 3000, Malvern Panalytical, UK), while their elemental compositions were determined via X-ray fluorescence spectroscopy (XRF, ZSX-Primus II, Rigaku, Japan). The surface micromorphology of the ceramic support was characterized using a benchtop scanning electron microscope (TM3000, Hitachi, Japan). The phase compositions of the fly ash and bauxite powders, as well as ceramic supports obtained at different sintering temperatures, were determined by X-ray diffraction (XRD, MiniFlex 600, Rigaku, Japan). The shrinkage was calculated by measuring the diameters of the disk supports before and after sintering at different temperatures using Vernier calipers (HengLiang, Shanghai, China). The shrinkage rate was calculated using the following equation:

(1) $$\varphi =\frac{D_{0}-D_{S}}{D_{0}}\times 100\%$$

where *φ* is the shrinkage of the ceramic support, and $D_{0}$, $D_{S}$ is the diameter (in mm) of the disk support before and after sintering, respectively. The porosity of the disk supports was measured via the Archimedes method using pure water as the liquid medium and calculated through the following equation:

(2) $$\varepsilon =\frac{m_{w}-m_{d}}{m_{w}-m_{s}}\times 100\%$$

where ε is the porosity; $m_{w}$ is the mass of the wet ceramic support, in g; $m_{d}$ is the dry mass, in g; and $m_{s}$ is the wet mass measured in water, in g. The pore size distribution of the supports was measured using a mercury porosimeter (Poremaster GT-60, Quantachrome Instruments, Middlesex, MA, USA). The pure water flux under the unit pressure of the disk supports was tested using a cross-flow filtration device and calculated according to Equations (3) and (4):

(3) $$F=\frac{V}{A\cdot t}$$

(4) $$J=\frac{V}{A\cdot t\cdot \Delta P}$$

where *V* is the volume of liquid on the permeate side, in L; *A* is the effective filtration area of the ceramic support, in m^2^; *t* is the filtration time, in h; Δ*P* is the transmembrane pressure, in bar; *F* is the pure water flux of the ceramic support, in L·m^−2^·h^−1^; and *J* is the pure water permeance of the ceramic support, in L·m^−2^·h^−1^·bar^−1^. The three-point bending strength of the rectangular column supports was measured using a universal testing machine (CMT-6203, SANS, China) with at least five samples for each test and calculated according to the following equation:

(5) $$\sigma =\frac{3Fa}{2bc^{2}}$$

where *σ* is the three-point bending strength of the ceramic support, in MPa; *a* is the length of the ceramic support, in mm; *b* and *c* are the width and height of the ceramic support, in mm; and *F* is the fracture force of the ceramic support, in N.

Ceramic supports A40-1200 and A40-1300 were placed in NaOH (10 wt%) and H_2_SO_4_ (20 wt%) solutions heated to 100 °C in an oil bath, and they exposed for 8 h; a set of samples was taken every 2 h. After 8 h of corrosion, the samples were soaked in 3.65 wt% HCl or 4 wt% NaOH solutions for 30 min to neutralize the residual alkali or acid on the surface of the samples, and then, all samples were rinsed with water; after that, the samples were placed in an oven at 70 °C for 12 h to completely remove the residual moisture. The dried supports were used to test the three-point bending strength and mass loss after corrosion. The mass loss rate was calculated using the following equation:

(6) $$\eta_{m}=\frac{m_{0}-m_{c}}{m_{0}}\times 100\%$$

where $\eta_{m}$ is the mass loss after corrosion of the ceramic support; $m_{0}$ is the mass of ceramic support before corrosion, in g; and $m_{c}$ is the mass of ceramic support after corrosion, in g.

## 3. Results and Discussion

### 3.1. Characterization of Raw Materials

**Figure 1:**
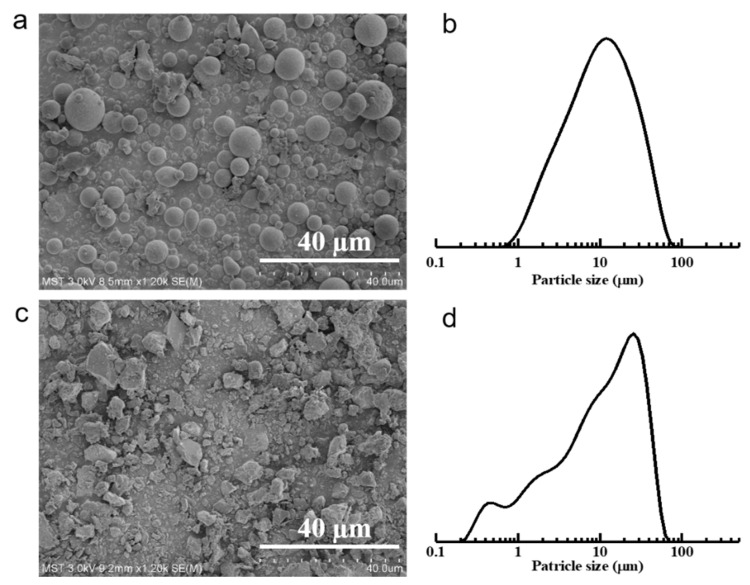
Morphologies and particle size distributions of fly ash (**a**,**b**) and bauxite (**c**,**d**).

The detailed compositions of fly ash and bauxite analyzed by XRF are listed in Table 2. The materials consisted mainly of Al_2_O_3_ and SiO_2_, with a total proportion of approximately 80 to 90 wt%. Other metal oxides can aid in reducing the sintering temperature because of their low melting points. The morphologies and size distributions of the fly ash and bauxite powders are shown in Figure 1. Fly ash particles were spherical, with a particle size range of 0.7–80 µm and an average size of 10 µm. Bauxite particles were irregularly shaped, with a particle size range of 0.2–70 µm and an average size of 25 µm.

**Figure 2:**
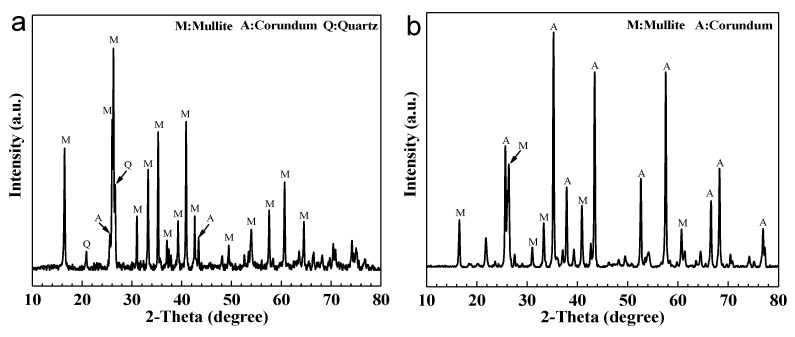
XRD patterns of fly ash (**a**) and bauxite (**b**).

The XRD patterns of fly ash and bauxite are shown in Figure 2. The crystalline phases of fly ash were mainly mullite, corundum, and quartz phases (Figure 2a), in agreement with the literature [34,35]. The mullite existing in fly ash is primary mullite, which is formed from aluminosilicate clay minerals during the combustion process of raw coal [36]. The crystalline phases of bauxite consisted mainly in mullite and corundum phases (Figure 2b); the latter was present in a relatively high amount. Therefore, when bauxite was added to fly ash as an aluminum source, it reacted with the fly ash quartz phase to form a mullite phase, which could enhance the performance of the ceramic supports.

### 3.2. Effect of Bauxite Doping on the Micromorphologies of Ceramic Supports

**Figure 3:**
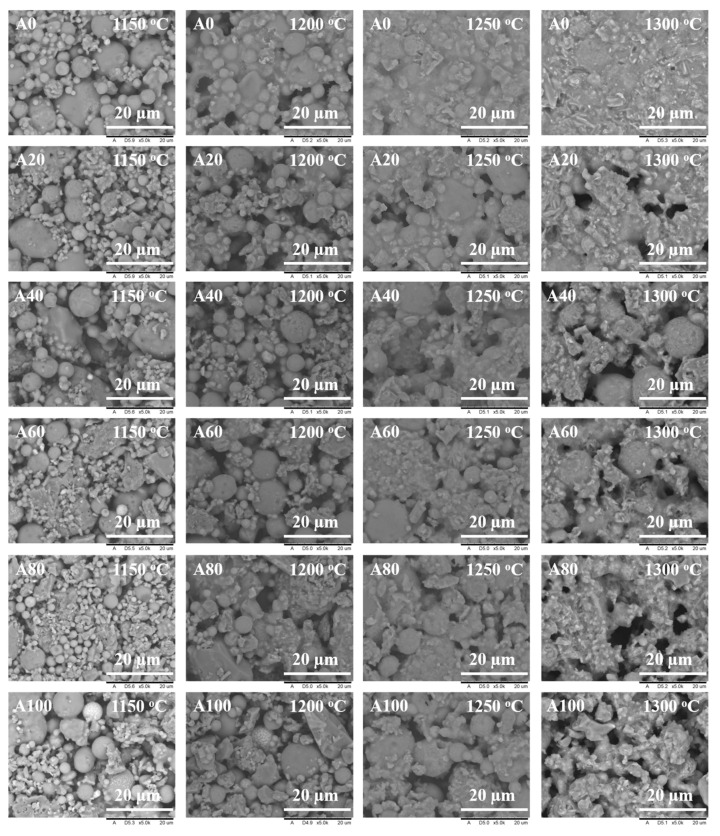
Microstructures of fly ash supports obtained by sintering at different temperatures with different bauxite contents.

The effects of bauxite addition on the micromorphologies of the ceramic supports sintered at different temperatures were studied, as shown in Figure 3. In the absence of bauxite, the A0 supports exhibited significant densification with an increase in the sintering temperature from 1150 to 1300 °C. Compared with the A20 to A100 supports, the A0 supports had a lower porosity, especially when the sintering temperature was above 1200 °C. When the sintering temperature was 1150 °C, the A0 supports exhibited only some initial neck connections between particles, suggesting a low degree of sintering. The same effect was observed even after adding bauxite (A20-A100 supports), indicating that in general, 1150 °C was not a suitable sintering temperature. When the sintering temperature increased to 1200 °C, the fly ash in the A0 supports melted and thus occupied the voids among particles, resulting in partial densification. However, the FESEM images of the bauxite-containing ceramic supports (A20 to A100) did not show a densified structure. With sintering temperatures of 1250 and 1300 °C, the A0 supports were very dense, and no pore structure could be observed. However, those containing bauxite (A20 to A100) showed strong neck connections and porous structures. These FESEM results demonstrate that the sintering temperature of pure fly ash supports should be adjusted in the narrow temperature range between 1150 and 1200 °C; however, the addition of bauxite can expand such temperature range up to 1300 °C.

### 3.3. Effect of Bauxite Doping on the Phase Composition of Ceramic Supports

**Figure 4:**
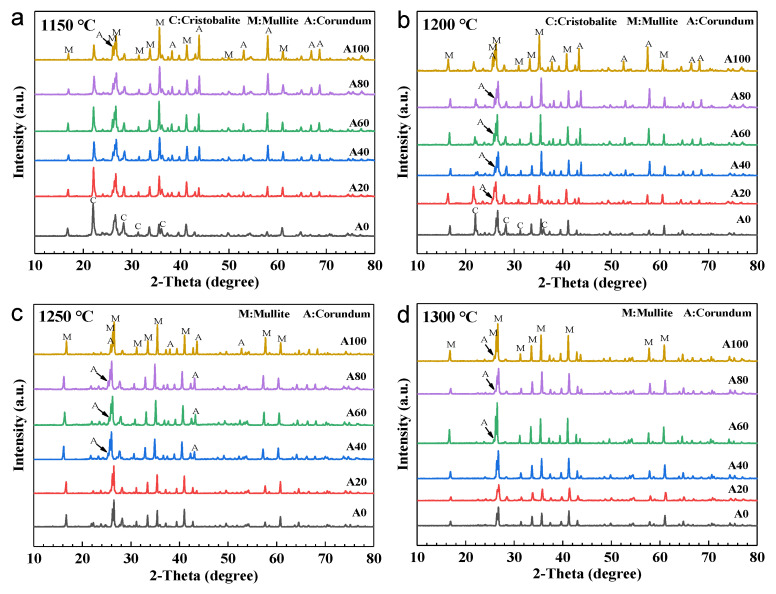
XRD patterns of fly ash supports with different bauxite contents sintering at 1150 °C (**a**), 1200 °C (**b**), 1250 °C (**c**) and 1300 °C (**d**).

In general, the resistance of fly ash supports to acid or alkali is determined by their phase composition. The phase of the supports sintered at different temperatures between 1150 and 1300 °C with different bauxite contents is shown in Figure 4. By applying a sintering temperature of 1150 °C, the main crystalline phases formed in the supports were cristobalite, mullite, and corundum (Figure 4a). The original quartz phase observed in the initial fly ash was no longer visible in the ceramic supports owing to its transformation into cristobalite at high temperatures [27]. As evidenced in the figure, the peak intensity of the cristobalite phase gradually decreases, while those of mullite and corundum gradually increase with increasing bauxite content. This indicates that the addition of bauxite to fly ash can promote the transformation of cristobalite into mullite. Upon further increasing the sintering temperature to 1200 °C (Figure 4b), cristobalite and corundum phases were still observed in the samples, indicating that the mullitization reaction was incomplete.

When the sintering temperature reached 1250 and 1300 °C (Figure 4c,d), the characteristic cristobalite peaks were no longer observed in the A20 to A100 supports, indicating that high temperatures can promote a complete mullitization reaction between cristobalite and corundum [37]. However, cristobalite was also no longer observed in the A0 supports; this was due to the presence of CaO and Na_2_O in the fly ash, which react with cristobalite to form a glass phase at high temperatures [38]. In the Figure 4c and d, the peak intensity of the mullite phase gradually increases with increasing bauxite content. Hence, by adding bauxite to the fly ash, the cristobalite phase reacts at high temperatures to form mullite instead of a glass phase.

### 3.4. Effect of Bauxite Doping on the Pore Structure of Ceramic Supports

**Figure 5:**
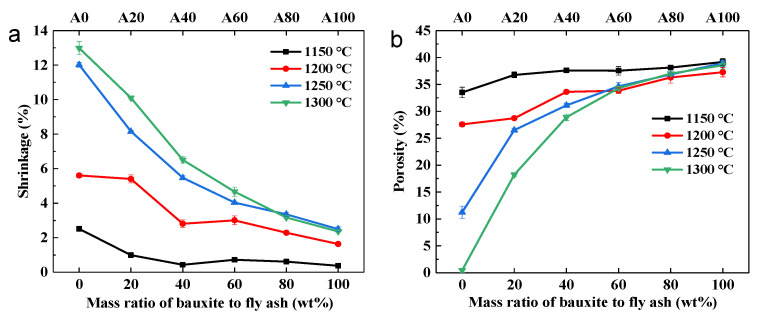
Shrinkage (**a**) and porosity (**b**) of fly ash supports obtained by sintering at different temperatures with different amounts of bauxite.

The effects of different bauxite contents on the shrinkage and porosity of the ceramic supports obtained by sintering at different temperatures are shown in Figure 5. In Figure 5a, the shrinkage of the supports clearly decreases with the increase in bauxite doping and is more evident at high temperatures. At a sintering temperature of 1150 °C, the shrinkage of the fly ash support without bauxite doping was 2.5% and show a slight decrease after bauxite doping. When the sintering temperature reached 1300 °C, the shrinkage rate decreased from 13% to 2.5%, with an increase in bauxite doping, indicating that the presence of bauxite can effectively inhibit the densification of fly ash.

The variation of support porosity as a function of bauxite doping is shown in Figure 5b: with an increase in bauxite content, the porosity of the supports increased, following an opposite trend to that observed for the shrinkage (Figure 5a). At a sintering temperature of 1150 °C, all samples exhibited high porosity. However, when the sintering temperature reached 1300 °C, the porosity of the undoped fly ash support (A0) was almost zero, indicating that the samples were almost completely densified at this time. As the bauxite doping increased, the porosity increased gradually, reaching the same level as that observed at 1150 °C for A100. Interestingly, the increase in porosity concerned mainly samples A20 and A40.

**Figure 6:**
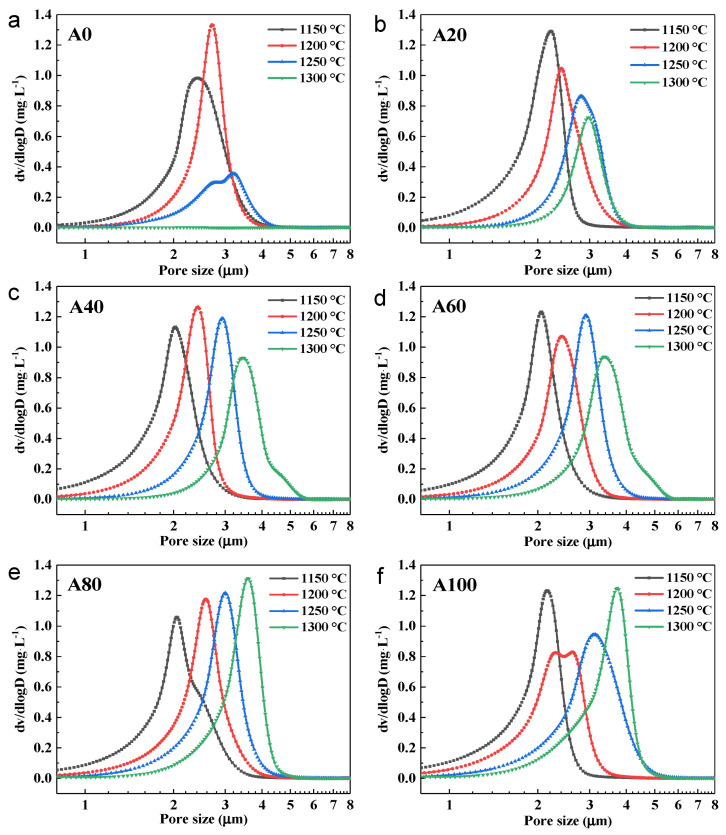
Pore size distribution of supports sintered at different temperatures for A0 (**a**), A20 (**b**), A40 (**c**), A60 (**d**), A80 (**e**), and A100 (**f**).

The effects of bauxite content and sintering temperature on the pore size distributions of fly ash/bauxite supports were investigated at different sintering temperatures between 1150 and 1300 °C; the results are shown in Figure 6. 

In the A0 supports (Figure 6a), the average pore size increased from 2.4 to 3.2 µm with increasing sintering temperature (from 1150 to 1250 °C). However, the porosity gradually decreased, as shown by the larger pore size but relatively smaller number of pores, which is indicative of particle growth. When the sintering temperature was 1300 °C, no pore structure could be observed, indicating that the A0 supports were completely densified, in agreement with the FESEM images.

For supports A20 to A100, the pore size increased with increasing sintering temperature. This is consistent with the trend observed in the A0 supports, where a high sintering temperature promoted particle growth. However, the sintering temperature did not have any clear adverse effects on the porosity of the A20 to A100 supports. In addition, when the sintering temperature was 1250 and 1300 °C, the A20 to A100 supports exhibited a narrower pore size distribution than A0. This demonstrates that the addition of bauxite can endow fly ash supports with a narrow pore size distribution while maintaining a high porosity. It is clear that the pore size distributions were determined by the sintering temperature and the amount of bauxite added.

### 3.5. Effect of Bauxite Doping on the Performance of Ceramic Supports

**Figure 7:**
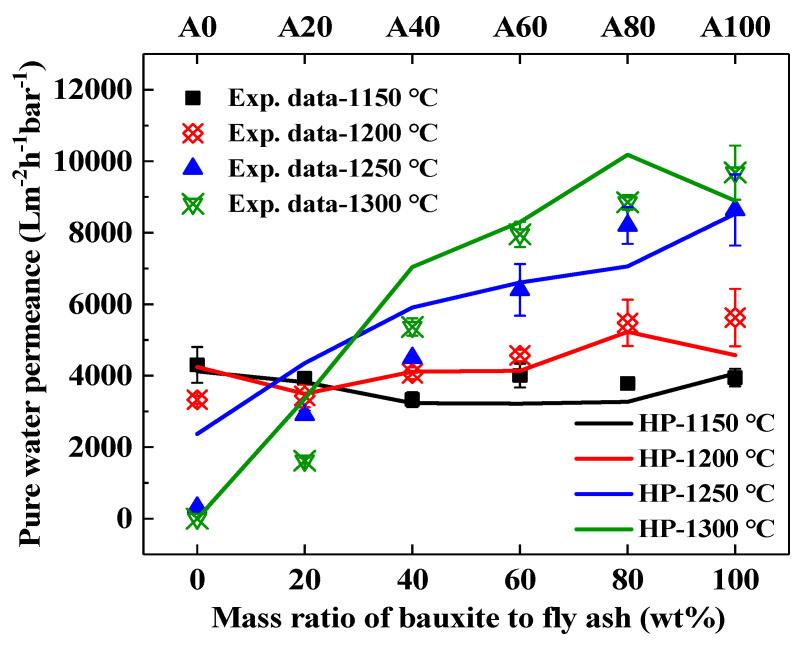
Experimental and calculated pure water permeance of fly ash supports obtained by sintering at different temperatures with different bauxite contents.

In general, high bending strength and high permeance to pure water are desirable properties for ceramic supports. The experimental and calculated pure water permeance of fly ash supports obtained by sintering at different temperatures with different bauxite contents are shown in Figure 7. The experimental data of the support were obtained using the Equations (3) and (4). The calculation value of the support was using the Hagen–Poiseuille equation [6]. The results can be seen that the trend of the calculation value is consistent with that of the experimental data.

(7) $$F=\frac{\Delta P\cdot d_{m}^{2}\cdot \varepsilon }{32\mu \cdot L\cdot \tau }$$

where *F* is the pure water flux, ΔP is the transmembrane pressure, $d_{m}$ is the pore size, ε is the porosity, μ is the viscosity of pure water, L is the thickness of the support, and τ is the tortuosity factor.

The pure water permeance of the A0 supports decreased from 4300 to 0 L·m^−2^·h^−1^·bar^−1^ for an increase in sintering temperature from 1150 to 1300 °C, as shown in Figure 7. This phenomenon is in a direct relationship with the porosity of the pure fly ash supports, which decreases with an increase in sintering temperature (from 0.33 to 0.004); therefore, the resistance increases dramatically during water filtration. When considering a specific temperature, we can observe an increase in pure water permeance with increasing bauxite doping. This is consistent with the variation in porosity, as the permeance is proportional to the porosity.

**Figure 8:**
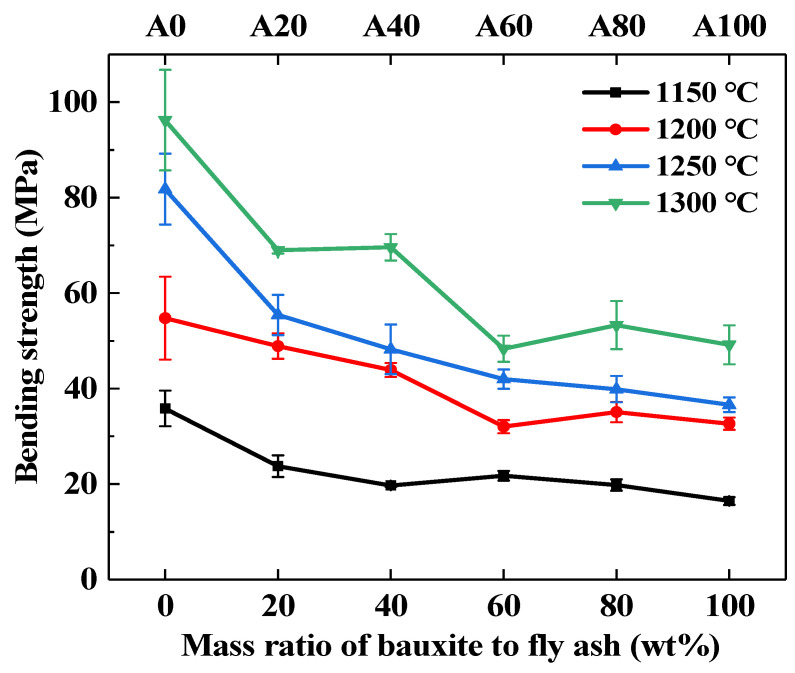
Three-point bending strength of fly ash supports obtained by sintering at different temperatures with different bauxite contents.

The bending strengths of the fly ash supports obtained with different sintering temperatures and bauxite contents are shown in Figure 8. The bending strength was proportional to the sintering temperature for each bauxite/fly ash ratio. For the A0 support, an increase in the sintering temperature determined an increase in the bending strength from 35 to 96 MPa. This represents a substantial increase and is mainly related to the melting of the fly ash particles at 1170 °C [39]. This, in turn, promoted the connectivity among fly ash particles and increased the interparticle bending strength. Doping with bauxite led to a slight decrease in bending strength following a trade-off effect between the bending strength and water permeance; this aspect is important and should be taken into account when choosing the appropriate bauxite doping in practical applications.

### 3.6. Corrosion Resistance of Optimized Ceramic Supports

**Figure 9:**
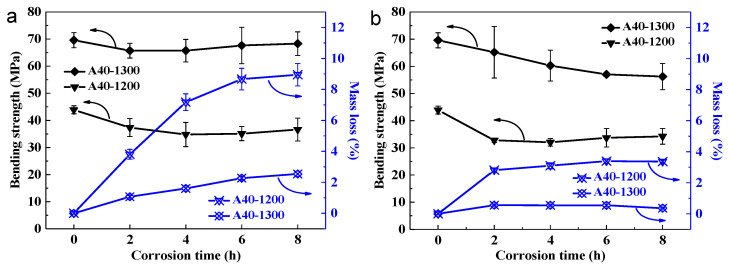
Bending strengths and mass loss rates of A40 samples sintered at 1200 and 1300 °C, before and after corrosion in 10 wt% NaOH (**a**) and 20 wt% H_2_SO_4_ (**b**) solutions at 100 °C.

The corrosion resistance of a ceramic membrane determines its range of applications. In this study, the A40-1200 and A40-1300 supports were tested to characterize the corrosion resistance of the fly ash support (see details in Section 2.3). The bending strengths and mass loss rates of the two supports before and after acid or alkali corrosion are shown in Figure 9. Here, the bending strength of A40-1200 decreases gradually with the increase in corrosion time in the NaOH solution; the bending strength approaches 34 MPa when the corrosion time is 8 h. However, for A40-1300, the decline is less marked, and the bending strength is still 65 MPa even after 8 h of alkali corrosion. To confirm the stability of their performance, the mass loss rates of the samples were determined, as shown in Figure 9a. As the corrosion time increased, the mass loss rate of A40-1200 increased gradually, reaching 8.95% after 8 h of corrosion. In contrast, the mass loss rate of A40-1300 presented a smoother variation and it was only 2.54% after 8 h of corrosion, which is consistent with the results of bending strength tests.

The bending strength and mass loss rate of the ceramic supports before and after acid corrosion are shown in Figure 9b. The bending strength of A40-1200 decreased from 44 to 32 MPa, and the strength loss was substantially concentrated in the first 2 h of corrosion. Sample A40-1300 still exhibited a strength of 56 MPa after 8 h of corrosion, and its value remained high. Figure 9b also shows the mass loss rates of the samples after acid corrosion: the maximum mass losses for A40-1200 and A40-1300 after 8 h of corrosion were 3.39% and 0.56%, respectively.

A comparison of the properties of the supports prepared in this work with those reported in the literature is shown in Table 3, evidencing that the low-cost ceramic supports obtained from fly ash and bauxite can be prepared at lower sintering temperatures. In addition, these supports exhibit a very stable performance even when corroded in acidic and basic environments at high temperatures. Hence, such low-cost supports can be employed in industrial applications under harsh conditions.

## 4. Conclusions

Fly ash and bauxite were used as raw materials to prepare ceramic supports. Bauxite served as the aluminum source to promote mullitization during sintering and increase the corrosion resistance of fly ash supports. The effects of the sintering temperature and amount of bauxite on the performance of the fabricated supports were thoroughly investigated. With an increasing bauxite doping, the shrinkage of the supports decreased while the porosity increased. The pore size of the supports decreased slightly with an increase in bauxite content, but the trend was not definite. In the supports having the same bauxite content, the pore size would increase at higher sintering temperatures. At 1300 °C and 40 wt% bauxite, we manufactured a fly ash support with a pure water permeance of 5.36 m^3^·m^−2^·h^−1^·bar^−1^ and bending strength of 69.6 MPa. The corrosion tests and comparison with the literature demonstrated the good resistance of this support to acid and alkali. Therefore, the supports prepared in this work can be employed in practical applications where resistance to harsh conditions is necessary.

## Figures and Tables

**Table 1:** Detailed compositions of ceramic supports. ^1^ The concentration of the PVA solution is 10 wt%.

Sample Code	Fly Ash (g)	Bauxite (g)	Glycerol (g)	PVA (g) ^1^
A0	100	0	4.0	3.0
A20	100	20	4.8	3.6
A40	100	40	5.6	4.2
A60	100	60	6.4	4.8
A80	100	80	7.2	5.4
A100	100	100	8.0	6.0

**Table 2:** Compositions of fly ash and bauxite (wt%). * Other represents the sum of other oxides with lower content, including SrO, ZrO_2_, CeO_2_, MnO, and V_2_O_5_.

	Al_2_O_3_	SiO_2_	CaO	C	Fe_2_O_3_	TiO_2_	K_2_O	MgO	P_2_O_5_	Na_2_O	Other *
Fly ash	39.35	42.88	4.09	3.84	3.63	1.55	1.22	0.61	0.54	0.29	2.00
Bauxite	71.89	18.98	0.34	0.69	3.02	3.21	0.48	0.12	0.22	0.04	1.01

**Table 3:** Comparison of corrosion resistance between the supports fabricated in this study and existing literature.

Raw Materials	Sintering Temperature	Bending Strength	Pore Size	Corrosion Test		Mass Loss/Bending Strength after 8 h Acid Corrosion	Mass Loss/Bending Strength after 8 h Alkali Corrosion	Ref.
	(℃)	(MPa)	(μm)	Acid	Alkali	%/MPa	%/MPa	
Cordierite, kaolin	1380	31.03	8.66	20 wt% H_2_SO_4_, 105–107 °C	10 wt% NaOH, 105–106 °C	17 N/A	12 27	[40]
Alumina, kaolin	1620	87.02	2.96	20 wt% H_2_SO_4_, 105–107 °C	10 wt% NaOH, 105–107 °C	1 78	23 15	[41]
Alumina, rice husk	1450	N/A	138	20 wt% H_2_SO_4_, 110 °C	10 wt% NaOH, 110 °C	0.95 N/A	2 N/A	[42]
Alumina, sugarcane bagasse	1450	N/A	84	20 wt% H_2_SO_4_, 110 °C	10 wt% NaOH, 110 °C	1.6 N/A	1 N/A	
Alumina, carbon black, sawdust, HEC, TiO_2_	1500	46.2	2.42	20 wt% H_2_SO_4_, 80 °C	10 wt% NaOH, 80 °C	0.44 N/A	0.39 N/A	[43]
Alumina, silica	1550	51.1	3.1	20 wt% H_2_SO_4_, 80 °C	10 wt% NaOH, 80 °C	0.20 48	2.0 47	[44]
	1550	49.7	3.1	20 wt% H_2_SO_4_, 80 °C	10 wt% NaOH, 80 °C	0.30 47	1.9 43	
Fly ash, bauxite	1300	69.6	3.4	20 wt% H_2_SO_4_ 100 °C	10 wt% NaOH, 100 °C	0.56 56	2.54 65	**This work**
